# Cats: The New Challenge for Rabies Control in the State of Yucatan, Mexico

**DOI:** 10.3390/pathogens13100907

**Published:** 2024-10-16

**Authors:** Aurea Mariana Salgado-Cardoso, José Ignacio Olave-Leyva, Ivonne Morales, Alvaro Aguilar-Setién, Irma López-Martínez, Nidia Aréchiga-Ceballos

**Affiliations:** 1 Instituto de Diagnóstico y Referencia Epidemiológicos, Dirección General de Epidemiología, Secretaría de Salud, Francisco de P. Miranda 177, Colonia Unidad Lomas de Plateros, Alcaldía Álvaro Obregón C.P. 01480, Ciudad de México, Mexico; salgadocardoso6@gmail.com (A.M.S.-C.); irma.lopez@salud.gob.mx (I.L.-M.); 2 Instituto de Ecología Aplicada, Universidad Autónoma de Tamaulipas, División del Golfo 356, Libertad, Ciudad Victoria C.P. 87019, Tamaulipas, Mexico; a2203138004@alumnos.uat.edu.mx; 3 Instituto de Ciencias Agropecuarias, Área Académica de Medicina Veterinaria y Zootecnia, Universidad Autónoma del Estado de Hidalgo, Rancho Universitario Avenida Universidad km 1, Ex-Hacienda de Aquetzalpa, Tulancingo C.P. 43600, Hidalgo, Mexico; 4 Department of Infectious Disease and Tropical Medicine, Heidelberg University Hospital, Im Neuenheimer Feld 324, 69120 Heidelberg, Germany; ivonne.morales@uni-heidelberg.de; 5 Heidelberg Institute of Global Health, Heidelberg University Hospital, Im Neuenheimer Feld 324, 69120 Heidelberg, Germany; 6 Programa de Maestría y Doctorado en Ciencias de la Producción y de la Salud Animal, Unidad de Posgrado, UNAM, Edificio “B” Primer Piso Circuito del Posgrado, Ciudad Universitaria, Coyoacán C.P. 04510, Ciudad de México, Mexico; balantiopterix@gmail.com

**Keywords:** rabies, *Felis catus*, spillover

## Abstract

The growing population in Yucatan has led to the expansion of construction in the Mayan jungle for tourist spaces, residential areas, and agriculture. Recently, rabies cases in cats (*Felis catus*) have increased in the state. This study aimed to perform antigenic and genetic characterization of the rabies viruses in felines and to present the spatial distribution and environmental features of the areas where these cases were reported. The ArcGIS software and R were employed to generate maps depicting the geographic locations of rabies cases in cats. A total of nine feline rabies cases occurred during the period 2003–2022. Three antigenic variants were detected: dog-related RVV1 (*n* = 1); vampire bat variant RVV3 (*n* = 1); and the canine-originated atypical variant (*n* = 7). Cases reported in Merida (*n* = 4) and Muna (*n* = 4) were localized to urban areas, while Cuncunul (*n* = 1) was rural. This study highlights the concerning resurgence of rabies infections in cats, emphasizing the looming threat of its reintroduction in dogs should vaccination rates diminish. The genetic affinity between the atypical variant and the canine virus underscores the urgent need for vigilance in maintaining high vaccination coverage across all susceptible species.

## 1. Introduction

Rabies is a disease caused by neurotropic viruses of the genus *Lyssavirus* in the family Rhabdoviridae, it is transmissible to all mammals, and it is almost uniformly fatal [1].

Cross-species transmission (CST) occurs when a rabies virus variant infects non-reservoir species, leading to disease. The frequency of CST events results from a complex interplay of eco-epidemiological, genetic, and evolutionary factors conditioning the specific virus–host relationship [2,3,4,5]. CST is primarily driven by the rate of physical contact between species rather than their relatedness [6]. This contact rate can be affected by changes in wildlife migration patterns and the resulting increase in abundance in new regions, which, in turn, alters interspecific interactions [7,8].

While all rabies reservoirs are also vectors for the virus, not all vectors function as reservoirs. Cats (*Felis catus*), whether domestic, free-roaming, or feral, can effectively transmit the rabies virus (RABV). However, there is no perpetuation of cat-to-cat rabies transmission. In comparison, infected dogs typically act as the predominant reservoir. Furthermore, no unique feline rabies virus variant has been documented [9].

Although human rabies transmitted by cats is infrequent [10], the potential for CST from wild-living mammals to domestic cat populations increases the risk of transmission to humans. This risk is heightened due to the multidimensional connections between humans and animals within our societies [2,11,12,13]. According to the SIRVERA (Regional Information System for Epidemiological Surveillance of Rabies) database, in the last five years, there were 60 cases of human rabies in the Americas region. Among these, cats transmitted twelve (20%): three in Cuba, two in Colombia (one with rabies virus variant (RVV4), two in Brazil (RVV3), one in Argentina (RVV3), two in Peru, and two in Mexico [14].

In the United States of America (USA), cats stand out as the most reported rabid domestic animal, with reported cases consistently surpassing those of dogs since the 1990s [15]. The virus can be found in the saliva of rabid cats, leading to transmission to humans through bites. Similarly to dogs, cats may shed the virus up to 10 days before the onset of clinical signs [16].

Despite the Ministry of Health in Mexico administering around 18 million rabies vaccine doses yearly—80% allocated for canines and 20% for cats (Supplementary Materials) [17]—three human rabies cases transmitted by cats have been reported in the past years: one in the state of Quintana Roo in 2004 (RVV3), one in Nayarit in 2022 [18], and the most recent one in Quintana Roo in 2024 (RVV5 related to the common vampire bat *Desmodus rotundus* was detected).

**Figure 1:**
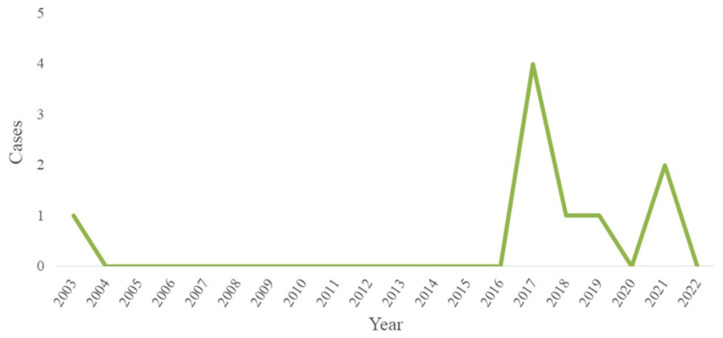
Time series of laboratory-confirmed rabies cases in cats in the state of Yucatan, from 2003 to 2022.

According to the 2020 census by the National Institute of Statistics and Geography (INEGI), Mexico’s pet population totaled 80 million, with 50% dogs, 20% cats, and 30% other species. This translates to four dogs and one cat for every ten inhabitants [19]. Significantly, the state of Yucatan holds the third-largest pet population nationally, with 2,074,423 pets. Of these, 44% are dogs, 23% are cats, and 33% belong to other species. Despite the administration of approximately 143,000 rabies vaccines annually in the state [19] (Supplementary Materials Figure S1), an increase in rabies cases among cats has been detected in the region (Figure 1).

Environmental disturbances, such as the growth of human settlements, agricultural expansion, and habitat fragmentation, are believed to influence the feeding behaviors and infection rates of wildlife species, such as the vampire bat [20]. In the Yucatan Peninsula, anthropogenic activities causing landscape fragmentation play a crucial role in ecological restoration and biodiversity preservation. The region’s growing population has driven human activity deeper into the Mayan Forest, resulting in the loss of 266,613 hectares over the past two decades at an average annual deforestation rate of 12,696 hectares [21]. Furthermore, the conversion of forestland to human settlements from 2001 to 2021 resulted in an average deforestation rate of 362 hectares per year [22,23].

These changes can facilitate the movement and introduction of animals to new areas, which, alongside the introduction of potential hosts and pathogens, significantly contribute to the emergence of infectious diseases [24]. Addressing the impacts of landscape fragmentation and human activities on both human and animal health necessitates the implementation of a One Health approach. This integrated methodology aims to sustainably balance and optimize the health of people, animals, and the environment by recognizing the interconnectedness and interdependence among human health, domestic and wild animals, plants, and the environment [25].

In this study, we conducted the antigenic and genetic characterization of RABV isolated from cats in the state of Yucatan between 2003 and 2022. Additionally, we presented the spatial distribution and features of the surrounding environment of these cases.

## 2. Materials and Methods

### 2.1. Samples

The total number of registered cases (*n* = 9) in cats from 2003 to 2022 in the state of Yucatan was obtained from the positive sample bank of the InDRE Rabies Laboratory. Each sample contained the brain and brain stem of each individual (Table 1).

### 2.2. Rabies Diagnosis

Rabies positivity was determined by the fluorescent antibody test [26], which uses a fluorescently labeled anti-rabies monoclonal globulin (Fujirebio, Diagnostics Inc., Seguin, TX, USA), in brain and brain stem tissue samples from all nine cases. Further confirmatory tests, including antigenic characterization, RT-PCR, and sequencing, were performed.

### 2.3. Antigenic Characterization

The antigenic characterization was performed using an indirect fluorescent antibody technique with a reduced panel of eight monoclonal antibodies (MAbs) (C1, C4, C9, C10, C12, C15, C18, and C19) donated by the Centers for Disease Control and Prevention, Atlanta, Georgia, as previously described [27,28]. This reduced panel can identify 11 reactivity patterns associated with different animals involved with rabies virus maintenance and transmission in Mexico and South America [29]. Antigenic characterization was applied directly to the brain smear; impressions were performed in an eight-well, 6 mm diameter slide. A positive reaction for each Mab (20 μL) was considered if more than 50% of the fluorescing foci brilliant apple green were observed.

### 2.4. Viral RNA Extraction

Fifty milligrams (mg) of brain tissue were macerated in lysis buffer: Tris 1 M, NaCl 5 M, MgCl2 0.5 M, and NP40 (Sigma). The buffer aids in the homogenization of brain tissues and the hypotonic lysis of the cells to free cytoplasmic RNA. The samples were subsequently centrifuged and the supernatant collected [30]. Finally, the QIAmp^®^ Viral RNA mini kit was used to collect viral RNA following the manufacturer’s instructions.

### 2.5. RT-PCR and Sequencing

Retrotranscription coupled to PCR was performed in 50 µL total volumes using the SuperScript Platinum kit following the manufacturer’s directions. Primers were added at a final concentration of 20 pmol/µL. The primers used were 550FW (5′-ATGTGYGCTAAYTGGAGYAC-3′), targeting positions 647–666 of the genome of the Challenge Virus Strain (CVS) [31], and 304 (5′-CGCTCTAGATTGACGAAGATCTTGCTCAT-3′), targeting positions 1514–1533 of the same strain [32], generating an 886 bp amplicon covering 65% of the coding sequence of the nucleoprotein gene. Thermocycler conditions consisted of 1 cycle at 42 °C for 60 min; 1 cycle at 94 °C for 5 min; 30 cycles at 94 °C for 30 s, 55 °C for 30 s, and 72 °C for 1 min; and 1 cycle at 72 °C for 5 min. PCR products were visualized by electrophoresis on the Agilent Bioanalyzer.

The DNA sequencing was performed with the BigDye Terminator v3.1 Cycle Sequencing kit^®^ employing the ABI PRISM^®^ 3130 xl Genetic Analyzer (Applied Biosystems, Foster City, CA, USA) according to the manufacturer’s recommendations. Sequences obtained in both senses were edited in BioEdit Sequence Alignment Editor 7.2.5 [33], and BLAST (Basic Local Alignment Search Tool) analysis (https://blast.ncbi.nlm.nih.gov/Blast.cgi, accessed on 4 April 2023) [34] from NCBI was performed. Edited files were converted to FASTA format to be used in the phylogenetic analysis.

### 2.6. Phylogenetic Analysis

The phylogenetic analysis involved 45 nucleotide sequences, including domestic and wild-living mammals from the Americas. The final dataset analyzed a region of 444 nucleotides. Multiple alignments of all sequences using the ClustalW Multiple Alignment application and phylogenetic analysis were performed with MEGA11 [35]. The evolutionary model was Kimura 2P + G using the neighbor-joining method.

### 2.7. Geographic Data

Advanced Geographic Information Systems (GIS) tools in ArcGIS and the National Institute of Statistics and Geography (INEGI) were used to obtain detailed geographic data at the municipal level for the state of Yucatan. This process included the overlaying of case data with the variable population size and vegetation type.

A comparative analysis of vegetation and land use in Yucatan was presented using georeferenced data from INEGI for 2001 [36] (“Vectorial Dataset of Land use and Vegetation, Scale 1:250,000, Series II. National Ensemble, scale: 1:250,000) and 2021 [37] (“Vectorial Dataset of Land Use and Vegetation”. Scale 1:250,000, Series VII. National Ensemble, scale: 1:250,000), and these data were compared with the occurrence of rabies cases in cats in Merida, Cuncunul, and Muna.

The population size for each municipality was based on the INEGI census of 2020. This analysis was performed in R.

## 3. Results

### 3.1. Rabies Diagnoses and Clinical–Epidemiological Features of Cases

Between 2003 and 2022, all nine samples from cats in the state of Yucatan tested positive for RABV via the FAT method. Notably, between 2016 and 2022, an increase in sample positivity was detected (Figure 1). The clinical-epidemiological characteristics of cases are described in Table 1.

### 3.2. Antigenic Variants Detected

Antigenic characterization revealed three virus variants. The oldest sample, dating back to 2003, corresponded to rabies virus variant (RVV) 1 (*n* = 1; 11%), the canine variant that circulated in dogs. Among the positive samples obtained between 2017 and 2021, seven (77%) corresponded to the unknown wildlife reservoir atypical RVV (see discussion of reservoir below). Additionally, the most recent sample from 2021 corresponded to RVV3 (*n* = 1; 11%), which is related to the common vampire bat (*Desmodus rotundus*) (Table 2).

### 3.3. Genetic Characterization and Phylogenetic Analysis

**Figure 2:**
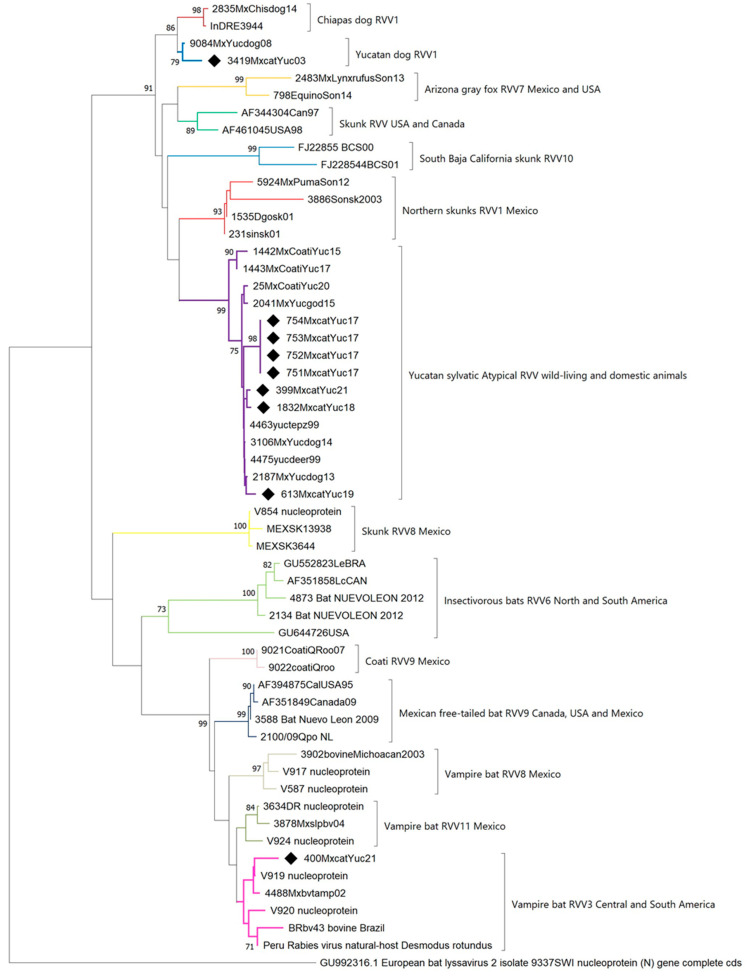
Phylogenetic reconstruction of the nine positive rabies cases in cats in Yucatan. The analysis was based on a dataset of 45 RABV partial nucleoprotein sequences belonging to terrestrial mammals and bats of the Americas. The neighbor-joining analysis was carried out using the evolutionary model Kimura 2P + G. ♦ Black diamonds indicate RABV isolated from cats in Yucatan.

For the phylogenetic analysis, a dataset of 45 sequences, each 444 bp in length and representative of rabies cases in the Americas, was included. Two main clades were identified that clustered the sequences of two rabies cycles, one related to terrestrial mammals and one related to bats (Figure 2). Phylogenetic reconstruction confirmed that the seven sequences corresponding to atypical RVV (751MxcatYuc17, 752MxcatYuc17, 753MxcatYuc17, 754MxcatYuc17, 1832MxcatYuc18, 613MxcatYuc19, and 399MxcatYuc21) clustered with a terrestrial cycle variant. This variant originated from the canine variant that was previously prevalent among dogs in Yucatan and became extinct due to mass vaccination programs.

The virus sequences from cases 3419MxcatYuc03 and 400MxcatYuc2021 were grouped according to the antigenic characterization that was previously carried out on the samples. The virus from the 2003 case (sequence 3419MxcatYuc03) clustered with viruses isolated from terrestrial mammals, specifically the dog rabies variant RVV1.

In this clade, the most recent case in dogs was detected in 2009, congruent with the history of elimination of this RVV1 in Yucatan. Since then, the atypical variant has been isolated most commonly from other terrestrial mammals (domestic and wild-living).

The virus sequence from the most recent case detected in 2021 (sequence 400MxcatYuc2021) clustered with the rabies cycle related to bats. In this clade, the sequences were related to the common vampire bat, the main reservoir of rabies in Yucatan in the bat-related cycle. So far, this is the only bat species that has been submitted for rabies diagnosis in the state, and the antigenic variants with bat origin detected in other terrestrial species submitted for rabies diagnosis in the state of Yucatan are related to this species.

### 3.4. Geographical Data

#### Spatial Distribution of Cat Rabies Cases

**Figure 3:**
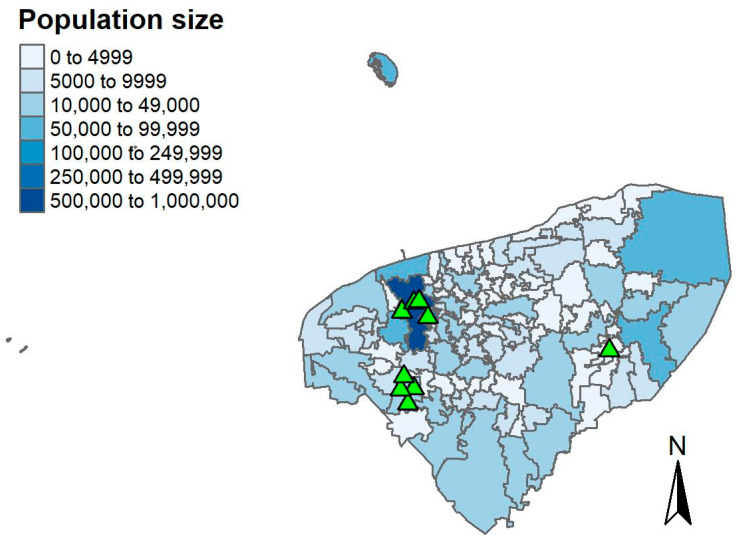
Geographic distribution of cat rabies cases (n = 9) in Yucatan at the municipality level, overlaid with population size. Rabies cases in cats are represented by the green triangles.

To gain insights into the spatial distribution of cat rabies cases, these were plotted at the municipality level. Out of the nine cases, four occurred in Muna, four in Merida, and one in the municipality of Cuncunul. Both Muna and Merida are predominantly urban areas, with populations of approximately 12,336 and 995,129 individuals, respectively. In contrast, Cuncunul is rural, with a population of around 1714 individuals (Figure 3).

**Figure 4:**
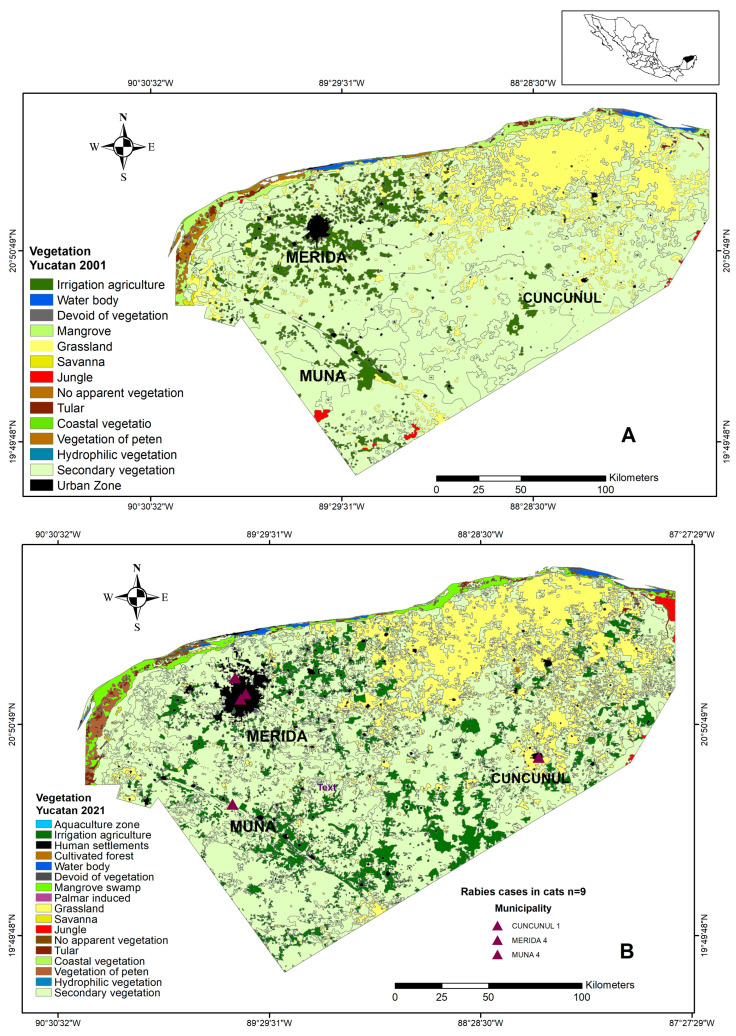
Maps of the changes in vegetation for 2001 and 2021. Panel (**A**): year 2001. Panel (**B**): Year 2021. Purple triangles show the georeferencing of the rabies cases registered in cats since 2003 in Yucatan, Mexico. (Modified from INEGI [36,37]) DATUM WGS84 geographic coordinates.

The maps in Figure 4 illustrate the expansion in urban areas, (irrigation) agriculture, and grassland from 2001 to 2021. Notably, urban areas around Merida increased in 2021, compared to 2001. Additionally, Muna experienced an extension of agricultural areas, while Cuncunul saw an expansion in grassland areas.

## 4. Discussion

Cats are explorative mammals [38] and efficient and abundant predators. Free-ranging cats can significantly impact ecosystems as generalist predators, exploiting a wide range of prey [39]. Since prey selection is correlated with prey availability [40,41], cats pose a threat to certain groups of animals, such as bats, birds, and rodents, both in human-modified and natural landscapes [39,42,43]. Consequently, cats are recognized as some of the most harmful predators to biodiversity among invasive or commensal species. In some cases, they contribute to the extinction of species on islands and cause significant wildlife mortality (especially birds and mammals), as observed in the USA and Australia [44,45,46].

In urban areas, domestic cats are the most abundant carnivores [46,47] and can prey on a large amount of wildlife each year [48]. Their predatory behavior stems from the heterogeneous conditions of free-ranging felines (owned or feral). A study conducted in Italy revealed that predation on bats mainly occurred in rural areas or areas characterized by single buildings interspersed with large vegetation patches where cats are often allowed to roam outdoors in rural or sparsely populated urban areas [42]. Conversely, owned cats in more densely populated urban areas were frequently kept indoors, potentially explaining the lower numbers of bats caught in such environments, despite the possibility of a higher absolute density of cats [49].

In this study, most of the infected cats were found in proximity to roads and were rescued by residents, indicating that they can be considered as free-roaming cats. Regarding the case from Cuncunul in 2021, residents reported that this cat had kittens and had established its shelter within the premises of a school, where it also sought refuge along-side other cats (Table 1). In general, cats displayed symptoms of aggression, inability to ingest water or food, and incoordination. Additionally, in three instances, there were reports of attacks on people and a pet dog. Although the cases occurred in an urban context, it is not known with certainty where they were infected. It is plausible that the point of CST may have been in a wilder environment. Since most of the cats were rescued, the cases were detected in an urbanized locality different from their origin, with the exception of the cats from Muna.

In our study, an important factor contributing to cats becoming potential vectors of the rabies virus is that none of the cases involved previously vaccinated animals. This is likely due to their young age (on average between two and four months old) and their status as free-roaming in the case of the adults. Consequently, it became necessary to apply post-exposure anti-rabies vaccination schedules to 32 persons, 18 of whom were bitten or scratched, along with canine pets that were directly or indirectly exposed to RABV-infected cats.

The phylogenetic analysis conducted suggests the presence of two epidemiological cycles of the rabies virus in the state of Yucatan, both associated with the wild-living cycle of rabies. One cycle involves hematophagous common vampire bats, which enzootically sustain the rabies virus in the state. As recently shown by Ortega-Sánchez et al. (2024), the state of Yucatan is among the Mexican states with the highest risk of rabies cases transmitted by vampire bats [50]. According to SIRVERA and the database of the Rabies Laboratory at InDRE, during the period covered by this study in the state of Yucatan, the following cases of rabies occurred: bovine (359), caprine (2), coati (4), deer (3), dog (44), equine (30), ovine (31), skunk (3), vampire bat (6), and non-determined wild-living mammals (7). Unfortunately, during the study period, the state of Yucatan recorded a case of human rabies transmitted by vampire bats in 2004 (RVV5) [14].

The other cycle corresponds to a mammalian terrestrial cycle. Currently, the atypical variant is believed to have its reservoir in certain wild-living species (skunks). Additionally, it has been detected in several mammals, such as the lowland paca (*Cuniculus paca*), the white-tailed deer (*Odocoileus virginianus*), and the white-nosed coati (*Nasua narica*) [51,52]. Evidence suggests that this antigenic variant originated from the dog variant that circulated in Yucatan but became extinct due to vaccination. It is now sustained and transmitted by some species of wild mammals, most likely skunks. Significantly, its canine origin poses a serious risk of reintroducing the virus into canine populations if vaccination coverage is not maintained [51,52].

Sociodemographic factors, such as poverty and population density, have been associated with the transmission of canine and human rabies in certain contexts [53,54]. INEGI data show that Yucatan’s urban and rural population grew from 1,658,210 in 2000 to 2,320,892 in 2020 [19], contributing to significant forestland loss. An estimated 8688 hectares are projected to be lost by 2024 [36,37]. This is significant, as forest cover has been implicated in rabies transmission by wildlife species, like skunks and raccoons [55].

In this study, we cannot rule out the possibility that CST events between bats and cats are influenced by factors different from those affecting transmission events between terrestrial mammals and cats. However, it is important to consider potential overlaps. For example, although bats are aerial mammals, their distribution, foraging behavior, and habitats are still closely tied to land cover [56,57].

Investigating the role of these factors in CST events could yield valuable insights, potentially informing future strategies for rabies control and prevention. Although the limited number of cases in this study prevented more in-depth analyses, the interrelatedness of these factors should be considered, as it may introduce additional complexity to models that aim to disentangle the effects of individual factors. Further investigations are warranted to better understand the underlying factors driving these dynamics.

Our finding of CST events in cats is a reminder of the importance of maintaining current vaccination status to protect animal health and prevent human exposure [2]. Vaccinating cats is often more challenging than vaccinating dogs, due to the difficulties associated with their capture and handling, which are compounded by their anatomy and temperament. In Mexico, particularly in provincial cities and towns like those in Yucatan, few cats live confined to apartments with regular contact with their owners. Most cats, even if fed by their owners, roam freely in gardens or natural areas. Unlike dogs, the majority of cats do not tolerate collars or harnesses, and few are accustomed to being transported in carriers. Handling them by hand poses a risk of bites and scratches, and capturing them outdoors is often difficult, due to their ability to hide in inaccessible places [58,59].

Understanding the mechanisms of host adaptation and interspecies transmission of RABV remains an important part of the ongoing goal to reduce and eliminate rabies [4]. A One Health approach to animal rabies prevention is crucial. This needs to be an interdisciplinary approach that considers host, pathogen, and environmental factors [60].

## 5. Conclusions

Our study sheds light on the emerging role of cats as a source of rabies virus transmission to humans in the state of Yucatan. We emphasize the critical importance of understanding CST dynamics from a One Health perspective to identify key drivers more precisely. Gaining these insights could pave the way for more targeted interventions.

## Figures and Tables

**Table 1:** Summary of the clinical manifestations and epidemiological data of the cat rabies cases.

#	Case	Year	Age/Sex ^a^	Vaccination Status	Type of Cat	Clinical Signs ^c^	Number of Humans Vaccinated	Number of Animals Vaccinated
1	3419	2003	NA	NA	NA	NA	NA	NA
2	751 ^b^	2017	2–4 months/female	Unknown	Rescued on the road between Muna and San Jose Tipceh	weakness, poor physical condition, lack of appetite	11 ^d^	All domestic animals in a 1 km radius
3	752 ^b^	2017	2–4 months/female	Unknown	Rescued on the road between Muna and San Jose Tipceh	weakness, poor physical condition, lack of appetite	11 ^d^	All domestic animals in a 1 km radius
4	753 ^b^	2017	2–4 months/female	Unknown	Rescued on the road between Muna and San Jose Tipceh	weakness, poor physical condition, lack of appetite	11 ^d^	All domestic animals in a 1 km radius
5	754 ^b^	2017	2–4 months/female	Unknown	Rescued on the road between Muna and San Jose Tipceh	weakness, poor physical condition, lack of appetite	11 ^d^	All domestic animals in 1 km radius
6	1832	2018	3 months/female	Unknown	Rescued; appeared inside the house	respiratory and nervous symptoms, eye and nasal discharges, incoordination of hind legs	3	All domestic animals in a 1 km radius
7	613	2019	3 months/male	Non-vaccinated	Rescued and adopted in two different houses	lack of appetite, aggressiveness, and nervousness, sensitivity to light, incoordination	6	All domestic animals in a 1 km radius
8	399	2021	1 year and 5 months	Non-vaccinated	Rescued; adopted in two different houses	weakness, lack of appetite, difficulty having a bowel movement, aggressiveness	9	All domestic animals in a 1 km radius
9	400	2021	Adult/female	Unknown	Free-roaming with kittens	aggressiveness	3	All domestic animals in a 5 km radius

NA = Not available. ^a^ Determined by the veterinarian. ^b^ Case belongs to the litter of kittens; therefore, we considered it as one outbreak with four cases. ^c^ As described in the clinical history. ^d^ Refers to the same group of eleven individuals who had contact with the kittens and were subsequently vaccinated.

**Table 2:** Summary of rabies cases in cats in the state of Yucatan.

#	Case	Year	Municipality	Antigenic RVV	Sequence Name	GenBank Accession Number
1	3419	2003	Merida	RVV1	3419MxcatYuc03	PP105582
2	751	2017	Muna	Atypical	751MxcatYuc17	PP105583
3	752	2017	Muna	Atypical	752MxcatYuc17	PP105584
4	753	2017	Muna	Atypical	753MxcatYuc17	PP105585
5	754	2017	Muna	Atypical	754MxcatYuc17	PP105586
6	1832	2018	Merida	Atypical	1832MxcatYuc18	PP105587
7	613	2019	Merida	Atypical	613MxcatYuc19	PP105588
8	399	2021	Merida	Atypical	399MxcatYuc21	PP105589
9	400	2021	Cuncunul	RVV3	400MxCatYuc21	PP105590

## Data Availability

All sequences generated in this study are available at Genbank with the following accession numbers: PP105582, PP105583, PP105584, PP105585, PP105586, PP105587, PP105588, PP105589, and PP105590.

## Supplementary Materials

The following supporting information can be downloaded at: https://www.mdpi.com/article/10.3390/pathogens13100907/s1, Figure S1: Vaccines applied in Mexico. Panel A. Shows the vaccines that were used in the vaccination campaigns that were carried out in the country. Panel B. Shows the number of vaccines applied in Yucatan. Despite the efforts, vaccines applied to cats represent only 6.9% of the total population of cats in the state of Yucatan in the year 2022.
