# Supplementary material for: Cats: The New Challenge for Rabies Control in the State of Yucatan, Mexico
Source: Pathogens. 2024 Oct 16;13(10):907. doi: 10.3390/pathogens13100907 (PMC11510174; doi:10.3390/pathogens13100907)
Supplement: Supplementary file 1 [file pathogens-13-00907-s001.zip › pathogens-3156856-supplementary.pdf]

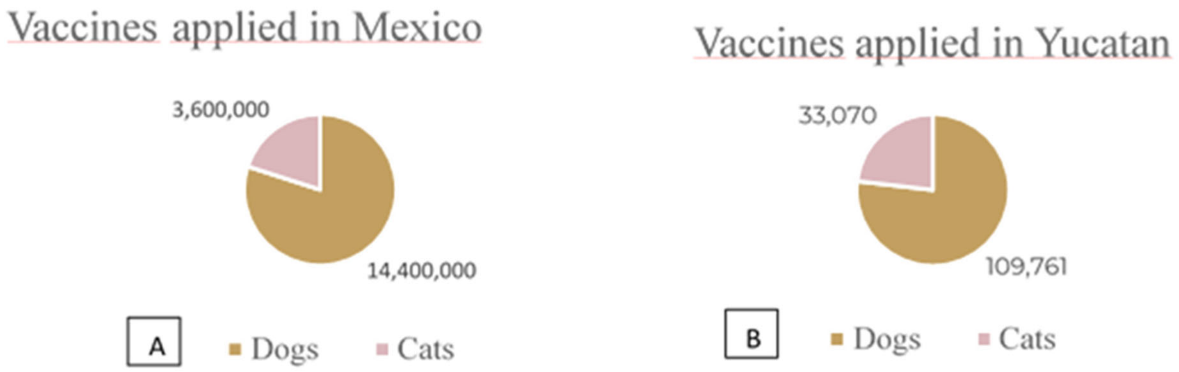

Figure S1. Vaccines applied in Mexico. Panel (**A**). Shows the vaccines that were used in the vaccination campaigns that were carried out in the country. Panel (**B**). Shows the number of vaccines applied in Yucatan. Despite the efforts, vaccines applied to cats represent only 6.9% of the total population of cats in the state of Yucatan in the year 2022.
